# Effects of leaf age during drought and recovery on photosynthesis, mesophyll conductance and leaf anatomy in wheat leaves

**DOI:** 10.3389/fpls.2023.1091418

**Published:** 2023-06-20

**Authors:** Eisrat Jahan, Robert Edward Sharwood, David T. Tissue

**Affiliations:** ^1^ School of Life and Environmental Sciences, The University of Sydney, Camden, NSW, Australia; ^2^ Hawkesbury Institute for the Environment, Western Sydney University, Hawksbury, Penrith, NSW, Australia; ^3^ School of Science, Western Sydney University, Hawksbury, Penrith, NSW, Australia; ^4^ Global Centre for Land-Based Innovation, Western Sydney University, Hawksbury, Penrith, NSW, Australia

**Keywords:** leaf internal conductance, leaf age, water stress, *Triticum aestivum*, leaf anatomy, recovery

## Abstract

Summary statement: Mesophyll conductance (*g*_m_) was negatively correlated with wheat leaf age but was positively correlated with the surface area of chloroplasts exposed to intercellular airspaces (*S*_c_). The rate of decline in photosynthetic rate and *g*_m_ as leaves aged was slower for water-stressed than well-watered plants. Upon rewatering, the degree of recovery from water-stress depended on the age of the leaves, with the strongest recovery for mature leaves, rather than young or old leaves. Diffusion of CO_2_ from the intercellular airspaces to the site of Rubisco within C_3_ plant chloroplasts (*g_m_*) governs photosynthetic CO_2_ assimilation (*A*). However, variation in *g*_m_ in response to environmental stress during leaf development remains poorly understood. Age-dependent changes in leaf ultrastructure and potential impacts on *g*_m_, *A*, and stomatal conductance to CO_2_ (*g*_sc_) were investigated for wheat (*Triticum aestivum* L.) in well-watered and water-stressed plants, and after recovery by re-watering of droughted plants. Significant reductions in *A* and *g*_m_ were found as leaves aged. The oldest plants (15 days and 22 days) in water-stressed conditions showed higher A and gm compared to irrigated plants. The rate of decline in *A* and *g*_m_ as leaves aged was slower for water-stressed compared to well-watered plants. When droughted plants were rewatered, the degree of recovery depended on the age of the leaves, but only for *g*_m_. The surface area of chloroplasts exposed to intercellular airspaces (*S*_c_) and the size of individual chloroplasts declined as leaves aged, resulting in a positive correlation between *g*_m_ and *S*_c_. Leaf age significantly affected cell wall thickness (*t*_cw_), which was higher in old leaves compared to mature/young leaves. Greater knowledge of leaf anatomical traits associated with *g*_m_ partially explained changes in physiology with leaf age and plant water status, which in turn should create more possibilities for improving photosynthesis using breeding/biotechnological strategies.

## Introduction

Wheat (*Triticum aestivum*) is counted among the ‘big three’ cereal crops after rice and maize, with an annual average harvest of *ca.* 750 million tonnes from 2016-2018 (FAO, 2019). Overall wheat is the staple food for almost half of the world’s population and wheat alone provides ≥ 20% of the calories and protein for the world’s population (Hawkesford et al., 2013). For our future predicted population of 870 million people by 2050, our global crop production needs to be increased by 60 – 110% to meet future demands (McGuire, 2012). The area farmed for wheat is constant over the last 50 years, so we must focus on continual improvements in yield to meet the rising demand (Evans and Lawson, 2020). In addition, anthropogenic changes in climate is a major environmental challenge which pose significant threats to water resources, crop production and food security (Hochman et al., 2017). In high latitude regions, precipitation is likely to increase, while it is predicted to decrease over large parts of the subtropics (IPCC, 2021). From 1980 to 2017, water-deficit decreased wheat and rice yields by 27% and 25%, respectively (Zhang et al., 2018). Consequently, it is expected that changes to water availability in future climates will directly impact photosynthetic carbon uptake, biomass production and crop yield (Sage and Kubien, 2007; Lawlor and Tezara, 2009; Alberth et al., 2011). To overcome those challenges, we need to understand the physiological responses of crops to water scarcity and their recovery after re-watering to build resilience within cropping systems and sustainably feed the future population.

Understanding the effect of leaf age on the components that underpin photosynthetic capacity is necessary to identify opportunities to improve production of key cereal crops in water limited environments (Wu et al., 2023). In addition, this knowledge will enable better estimates of the long-term carbon budget of the leaf and whole plant. Daily carbon gain is closely associated with photosynthetic capacity and photosynthetic rate (Reich et al., 1991; Gay and Thomas, 1995; Zhang et al., 2008).

A considerable amount of work has been done in relation to leaf age with different types of plants species. Clarke et al. (2021) reported for tobacco that photosynthetic rates (*A*) decreased as leaves aged and were lower in the canopy. Alonso-Forn et al. (2022) demonstrated in *Quercus ilex* the age-dependent adjustments of g*_m_* and *A* were correlated to changes in anatomy and photosynthetic biochemistry. Kitajima et al. (2002) reported a decline in photosynthetic capacity with increasing leaf age and position in two tropical tree species, *Cecropia longipes* and *Urera caracasana.* The age-related decline is not an uncontrolled physiological deterioration but is caused by redistribution of resources (such as nitrogen and carbon) from older to newer leaves (Hikosaka et al., 1994; Ackerly, 1996).

Crops consist of a population of leaves occupying different positions with different age structures and microhabitats within the canopy. For wheat, photosynthetic rates (*A*) for each leaf showed a short-term rise after ligule emergence, a plateau, and then a linear decline as the leaf aged (Rawson et al., 1983). An understanding of photosynthesis variation across different leaves is crucial for developing crop models to better predict where improvements in photosynthesis can be made; for example, rice (Yin and Struik, 2017). Therefore, greater understanding of the effects of leaf age on photosynthetic rate is important for crop production.

Photosynthetic rate (*A*) often has a strong positive correlation with mesophyll conductance (*g*_m_, the diffusion of CO_2_ from substomatal cavities to the carboxylation sites in the chloroplasts;Barbour et al., 2010; Giuliani et al., 2013; Jahan et al., 2014; Xiong et al., 2016; Ellsworth et al., 2018; Knauer et al., 2019; Gago et al., 2020; Jahan et al., 2021) and decreased *g*_m_ is considered to be one of the main factors involved in early age-induced photosynthetic decline (Niinemets et al., 2005; Flexas et al., 2007; Zhang et al., 2008). Conversely, Tosens et al. (2012) found *g*_m_ and *A* increased, and the CO_2_ drawdown from intercellular airspace to chloroplast (*C*_i_-*C*_c_) decreased, with increasing leaf age in *Populus tremula* L. Whitehead et al. (2011) did not find leaf age effects between current-year leaves and one-year old leaves on stomatal conductance (*g*_sc_) and *g*_m_ but there was a significant decline in *g*_m_ with increasing tree height in *Nothofagus solandrii* var. *cliffortiodes*.

Leaf anatomical traits such as leaf cell wall thickness, chloroplast spatial distribution, chloroplast to leaf area ratio, and mesophyll cell surface area exposed to intercellular airspace per unit of leaf surface area, are among the strongest determinants of *g*_m_ (Evans and von Caemmerer, 1996; Tosens et al., 2012; Giuliani et al., 2013; Tomas et al., 2013; Xiong et al., 2016; Evans, 2020). The surface area of chloroplasts exposed to intercellular airspace (*S*_c_) is often strongly positively related to *g*_m_ (Evans et al., 1994; Terashima et al., 2006; Evans et al., 2009; Tosens et al., 2012; Peguero-Pina et al., 2017; Carriquií et al., 2019; Clarke et al., 2021). The relative importance of leaf anatomy to the response of *g*_m_ as leaves aged is unclear, although Evans and Vellen (1996) found that while *g*_m_ declined as wheat leaves aged, there was no change in *S*_c_. In addition to these anatomical traits, metabolic factors, such as activity of aquaporin and carbonic anhydrase, may be involved in the regulation of *g*_m_ (Price et al., 1994; Hanba et al., 2004; Flexas et al., 2006a; Momayyezi and Guy, 2017; Ogeíe et al., 2018; Xu et al., 2019; Ermakova et al., 2021).

One of the well-known responses to water stress is stomatal closure, which increases the resistance to CO_2_ diffusion into leaves (Kaiser, 1987). The CO_2_ concentration inside the chloroplast may be further restricted by resistance inside the mesophyll (resistance through the liquid phase inside cells, *g*_liq_) and this resistance is also likely to increase in water-stressed leaves (Cornic and Massacci, 1996; Loreto et al., 1997: Evans and von Caemmerer, 2013). Mesophyll conductance (the diffusion of CO_2_ from substomatal cavities to the carboxylation sites in the chloroplasts) is a dynamic leaf trait and in particular, *g*_m_ was found to vary with water availability; *g*_m_ was lower under water stress for *Populus tremula* (Tosens et al., 2012), *Eucalyptus regnans*, *Solanum lycopersicum* and *Phaseolus vulgaris* (Warren, 2008), and rice (Wang et al., 2018). However, *g*_m_ was unaffected by drought for bell pepper (*Capsicum annuum* L.) and sugar beet (*Beta vulgaris* L.) (Delfine et al., 2001; Monti et al., 2006). In C_3_ leaves, water stress predominantly affects CO_2_ diffusion, generating decreases in both stomatal and mesophyll conductance without decreasing the biochemical capacity to assimilate CO_2_ (Flexas et al., 2004).

The recovery phase following rewatering is an important part of the overall plant physiological response to drought and may be dependent on the degree of water stress (Flexas et al., 2006b; Chaves et al., 2009; Lawlor and Tezara, 2009). The behaviour of *g*_m_ recovery depends on environmental conditions: in spring, *g*_m_ recovers rapidly (within 1-2 days) after rewatering while it remained low many days after re-watering in summer (Galle et al., 2009). Those researchers also found *g*_m_ initially declined with water stress, then recovered to control values during the acclimation period. The recovery after re-watering remains an important aspect of plant response to drought (Miyashita et al., 2005; Galle et al., 2007; Galmes et al., 2007; Peguero-Pina et al., 2018).

The combined effect of leaf age and water limitation on mesophyll conductance has been assessed in ash and oak trees (Grassi and Magnani, 2005), where water stress reduced *g*_m_ but the impacts were not confounded by leaf ontogeny. Moreover, Varone et al. (2012) worked on three different Mediterranean species under water stress, and observed that net assimilation rate decreased slowly in seedlings (1-year old) due to stomatal limitations, while in saplings (3 to 4-years old) it decreased more quickly and was mainly associated with *g*_m_. Clearly, there is still limited and partly conflicting information on dynamic modifications of *g*_m_ through leaf ontogeny, and on the long-term water deficit effects on *g*_m_ (Tosens et al., 2012).

The primary objectives of this study were to investigate the relationship between morphological and anatomical traits in well-watered, water-deficit, and re-watered wheat (*Triticum aestivum* L.) plants that affect physiological responses of *A*, stomatal conductance to CO_2_ (*g*_sc_) and *g*_m_ as leaves aged. The following hypotheses were tested: (A) *g*_m_ changes significantly as individual leaves aged and has a correlation with *S*_c_; (B) leaf age plays a role in the rate of change in *A* and *g*_m_ for water stressed plants; and (C) after rewatering, leaf age plays a role in the degree of recovery for *g*_m_.

## Materials and methods

### Plant material and growth conditions

*Triticum aestivum* L., cultivar ‘Tasman’, was used to address the role of leaf age on leaf ultrastructure and plant physiological response to water stress and water stress recovery. The cultivar ‘Tasman’ is one of the parents in a quantitative trait loci (QTL) mapping population studied in depth for Δ^13^C of leaf tissue by Rebetzke et al. (2009); the cultivar has low stomatal conductance to CO_2_ (*g*_sc_) and high leaf-intrinsic water-use efficiency (*A*/*g*_sw_) (Jahan et al., 2014).

#### Water stress experiment

In the first part of this study, plants were grown in a controlled-environment growth room at the University of Sydney, Centre for Carbon Water and Food (Camden, NSW, Australia). The environment inside the growth room was 25°C during the 14-h light period [photosynthetic photon flux density (PPFD) was 800 µmol m^-2^ s^-1^ at the top of the plants], and 17°C during the 10-h dark period, while relative humidity was maintained at 75% at all times. Seeds were planted in 6-L pots filled with commercial potting mix with slow-release fertilizer (Osmocote Exact, Scotts, NSW, Australia). Seedlings were thinned to one plant per pot after emergence. Pots were well-watered for five weeks after emergence. Water treatments started for half of the plants at the sixth week after emergence. At the temporary wilting point (7 days after the start of the drought treatment), total weights of pot and plants were recorded for all water-stressed pots, and this was taken as a target water content. A visual assessment of leaf wilting was used as an indicator of water status for water-stressed plants where the temporary wilting point was defined as the first day on which leaves of water-stressed plants wilted. The target water content was maintained gravimetrically thereafter to introduce a mild water stress to half the plants. The well-watered pots were well-watered throughout the experiment. Pots were moved and rotated routinely to minimize the effect of environmental heterogeneity within each growth room. Gas exchange measurements commenced in the fifth week, which was one week prior to the start of the water-stress treatment.

#### Water stress recovery experiment

In the second part of our experiment, plants of the same cultivar ‘Tasman’ of wheat (*Triticum aestivum* L.) were grown in a controlled-environment growth room, under the same environmental conditions as above, to assess the recovery of plants from the drought treatment. Plants were well watered up to 3 weeks after emergence. Then water was withheld until temporary wilting point, when water content was maintained gravimetrically as described above. Gas exchange measurements were made on the sixth week after emergence. On the seventh week after emergence, plants were re-watered by soaking pots overnight in a full bucket. Gas exchange measurements recommenced on the following day to assess the recovery of *g*_m_ after re-watering leaves of different ages (i.e. leaves were remeasured one day after rewatering, but one week after the initial measurements under drought conditions). A Scholander-style pressure chamber (115, Soil Moisture Equipment, Santa Barbara, CA, USA) was used to measure the water potential (Ψ_l_) at midday for well-watered and water stress plants on leaves of three different ages. Leaves were wrapped in plastic film and cut just above the ligule with a razor blade prior to sealing in the pressure chamber for measurement.

**Figure 1:**
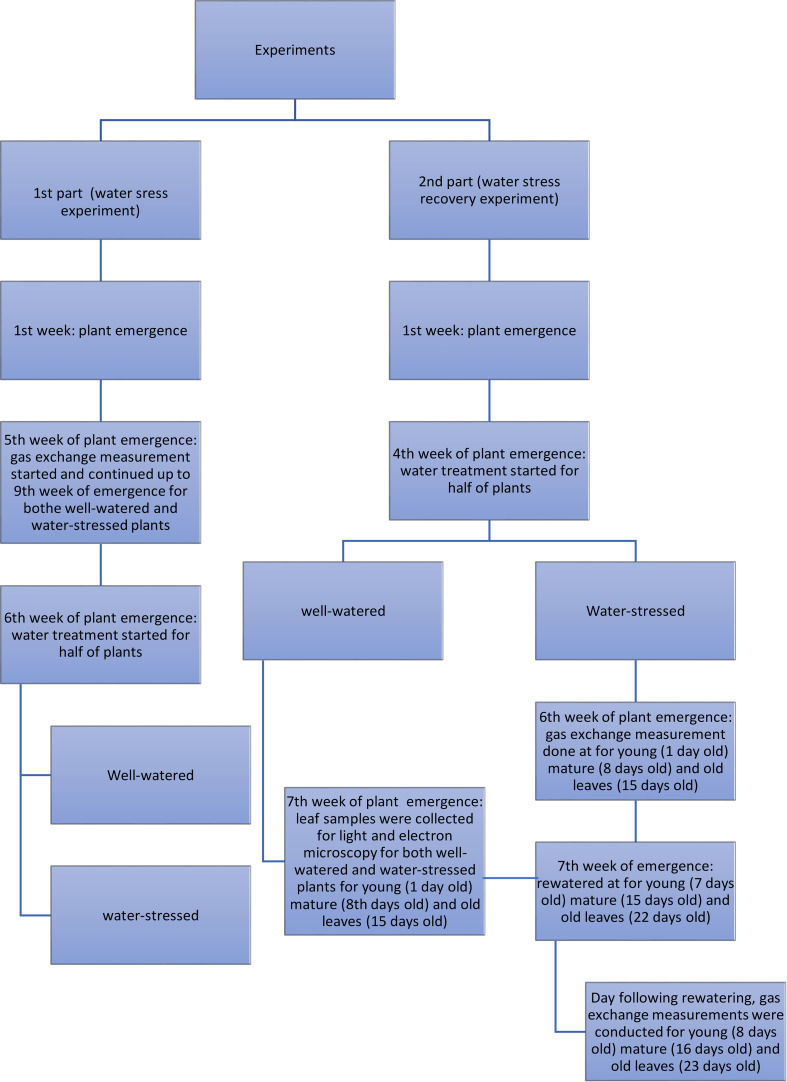
Scheme of the experimental design of the water stress and water stress recovery experiments.

A scheme of the experimental design of the water stress and water stress recovery experiments is presented in **Figure 1**.

### Estimation of g_m_

Two or three of the youngest fully expanded leaves per plant were selected for gas exchange measurements (weeks 5 to 9 after plant emergence). Leaves were labelled on the week of their full expansion (leaf number 5 on week 5, up to leaf 9 on week 9), and not by their position or tiller number. Under the experimental growth conditions used, leaves took between 5 and 8 days to expand, so that the ‘fifth leaf’ was 5-8 days older than the ‘sixth leaf’. Same-aged leaves from the same plant were placed side-by-side in a 6 cm^2^ (2×3) leaf chamber of a LI-6400XT portable photosynthesis system fitted with a red-blue light (LI-COR, Lincoln, NE, USA). During the time of measurement, all the plants were at the tillering phase except week 9, when the flowering heads began to emerge. During measurements, the leaf chamber was controlled at a CO_2_ mole fraction of 380 µmol mol^-1^, a leaf temperature of 25°C and irradiance of 1500 µmol m^-2^ s^-1^. The vapour pressure deficit inside the leaf chamber varied between 0.6 and 2.3 kPa, depending on *g*_sw_. Mesophyll conductance was estimated by combining leaf gas exchange measurements with measurements of carbon isotope discrimination using a tunable-diode laser absorption spectrometer (TDLAS, model TGA100A, Campbell Scientific, Inc., Logan, UT, USA) as described by Barbour et al. (2010) and Jahan et al. (2021).

When assessing the recovery of plants from the water stress, leaves were placed side-by-side in a 12 cm^2^ (2×6) leaf chamber (Li6400-11) attached to a LI-6400XT portable photosynthesis system (LI-COR, Lincoln, NE, USA) fitted with a red-green-blue light set to mimic the red-blue light source used in the first experiment (Li6400-18 RGB light source). The chamber environmental conditions were similar for all physiological measurements and represented light-saturated photosynthesis. Leaves were labelled based on age of their full expansion: young leaf is the newly fully expanded leaf (1 day old), mature leaf is one week older than young leaf (8 days old) and old leaf is one week older than mature leaf (15 days old), and not by their position or tiller number.

The value of *g*_m_ was calculated from the difference between predicted discrimination assuming infinite mesophyll conductance (Δ_i_) and measured discrimination (Δ_obs_), as described by Jahan et al. (2021) using equations developed by Evans et al. (1986) and Barbour et al. (2010), and including a ternary effect as described by Farquhar and Cernusak (2012). Average δ^13^C during the light period for a 7-day running average (δ^13^C*_CO2_*) was measured using a stable isotope cavity ring down laser (G11101-i, Picarro CA, USA). We used the average ambient growth δ^13^ *C_CO2_* values (-13.2 ‰) in the calculation of *g*_m_. The Picarro laser was calibrated as described by Thurgood et al. (2014). Here, values of fractionation factors were used in the calculation of *g*_m_ as described by Jahan et al. (2014); the fractionation associated with carboxylation was assumed to be 29‰, the fractionation during dissolution and diffusion through water was assumed to be 1.8‰, the fractionation associated with photorespiration was assumed to be 16.2‰, the fractionation occurring during diffusion through the leaf boundary layer was assumed to be 2.9‰ and the rate of day respiration was taken to be 0.8 µmol m^-2^ s^-1^.

### Light and electron microscopy

Leaves measured for gas exchange were analysed using microscopy to assess anatomical features as a function of leaf age and drought treatment. Leaf samples approximately 5 mm × 2 mm were cut for sectioning, avoiding major veins. Sections were fixed in a solution of 2.5% glutaraldehyde, 3% paraformaldehyde and 0.1 M phosphate buffer (pH 7.2) for 18 h period under vacuum. Samples were then fixed in 2% osmium tetroxide for 2 h, then further fixed using 2% uranyl acetate for 30 min. Samples were dehydrated using an ethanol series of 15, 30, 50, 70, 90, 95 and 100% and then embedded in LR-white resin series of 20, 40, 60, 80, 100% (London Resin Company, London, UK). Semi-thin leaf cross-sections of 0.5 µm for light microscope were cut using an ultra-microtome with glass tip blades, then sections were stained with toluidine blue.

Sections were viewed in bright field with a Leica DM6000B upright light microscope with phase contrast at 400× magnification. We used IMAGE J software (National Institute of Health, Bethesda, MD, USA) for analysis of images. Images were used to determine: (a) leaf thickness, (b) mesophyll thickness between the two epidermal layers (*t*_mes_), (c) the surface area of mesophyll cells exposed to the intercellular airspaces (*S*_mes_), (d) the surface area of chloroplast exposed to intercellular airspace (*S*_c_) and (e) chloroplast number per unit leaf area (*Z*, in number per m^2^). *S*_mes_, *S*_c_ and Z were calculated as described by Evans et al. (1994).


(1) $$S_{mes}=\frac{L_{mes}}{W}F$$

where *L_mes_* is the total length of mesophyll cells facing the intercellular airspace, *W* is the width of the section measured and *F* is the curvature correction factor (1.25) by Thain (1983). Then,


(2) $$S_{c}=\frac{L_{c}}{L_{mes}}S_{mes}$$

where, *L*_c_ is the length of chloroplast exposed to the intercellular airspace.


(3) $$Z=\frac{z}{L_{mes}}S_{mes}$$

where, *z* is the total number of chloroplasts in the section measured. The volume fraction of inter-cellular airspace (*fi*as) was calculated as described by Xiong et al. (2016),


(4) $$f_{ias}=\frac{S_{ias}}{S_{mes}}$$

where *S*_ias_ is the total cross-sectional intercellular airspace area.

Ultrathin cross- and paradermal sections of 70 nm were prepared by an ultramicrotome for use in the scanning transmission electron microscope (STEM). The sections for STEM were stained with lead citrate and mounted on glass with Eukitt (Electron Microscopy Sciences, Hatfield, PA, USA). Cell wall thickness (*t*_cw_), and chloroplast to outer cell wall distance were measured from STEM micrographs by using IMAGE J software.

### Statistical analysis

The effects of leaf age on physiological measures were assessed using two-factor analysis of variance (ANOVA); means were compared using Fisher’s unprotected least significant difference tests. The associations between *g*_m_ and *A*, between *g*_m_ and *g*_sc_, between *S_c_* and *g*_m_, and between *t_cw_* and *g*_m_ were assessed using linear regression and correlation. GenStat 14^th^ edition (VSN International Ltd, Hemel Hempstead, UK) was used for statistical analyses, and in all tests, results were considered statistically significant when *P<* 0.05.

## Results

### Leaf age effects

**Figure 2:**
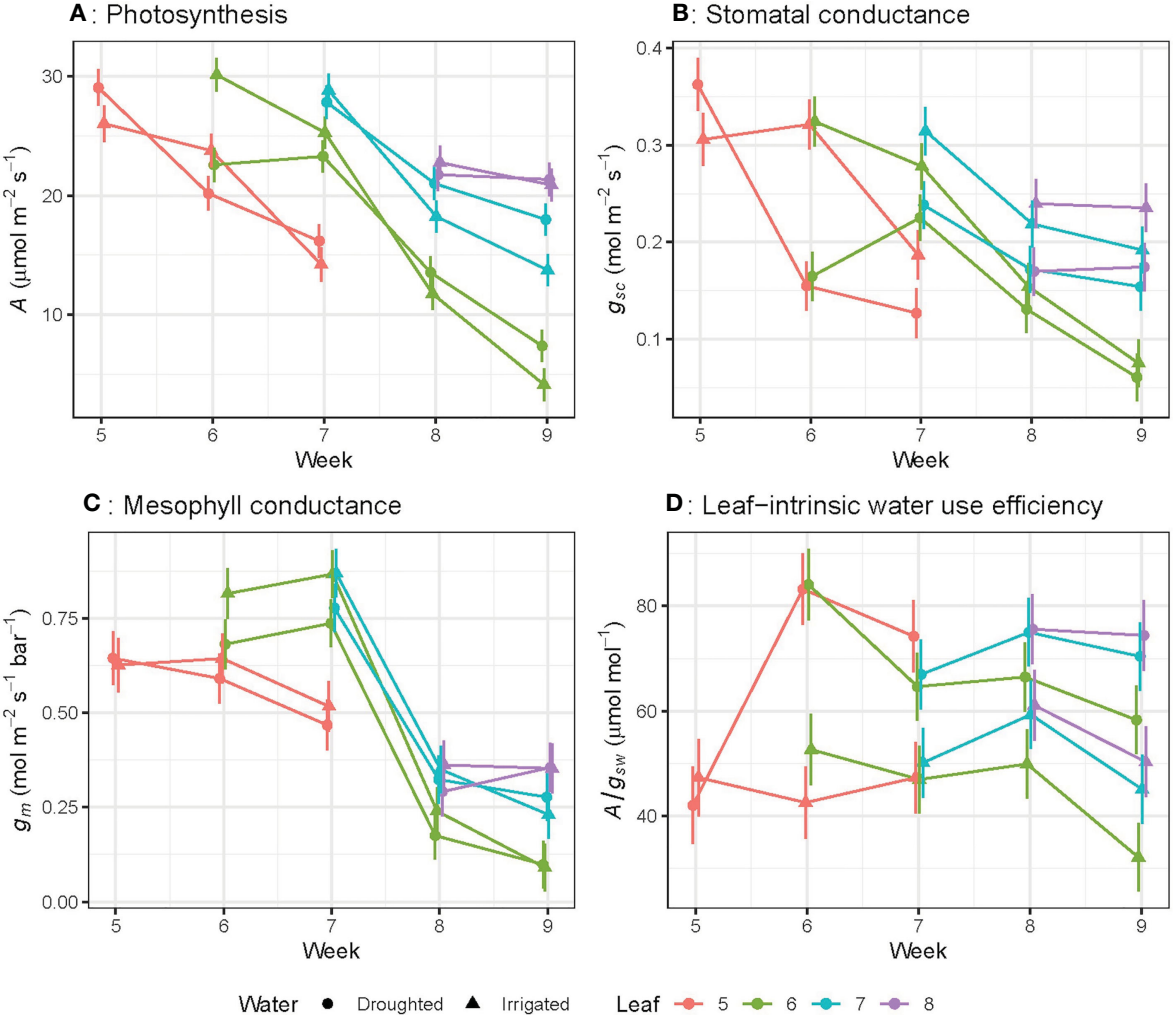
Light-saturated photosynthetic rate (A) **(A)**, stomatal conductance to CO2 (*g*_sc_) **(B)**, mesophyll conductance (*g*_m_) **(C)** and leaf-intrinsic water use efficiency (A/*g*_sw_) **(D)** for wheat leaves as they emerge and age from week 5 to week 9. The bar represents the average standard error, *n* = 5.

**Figure 3:**
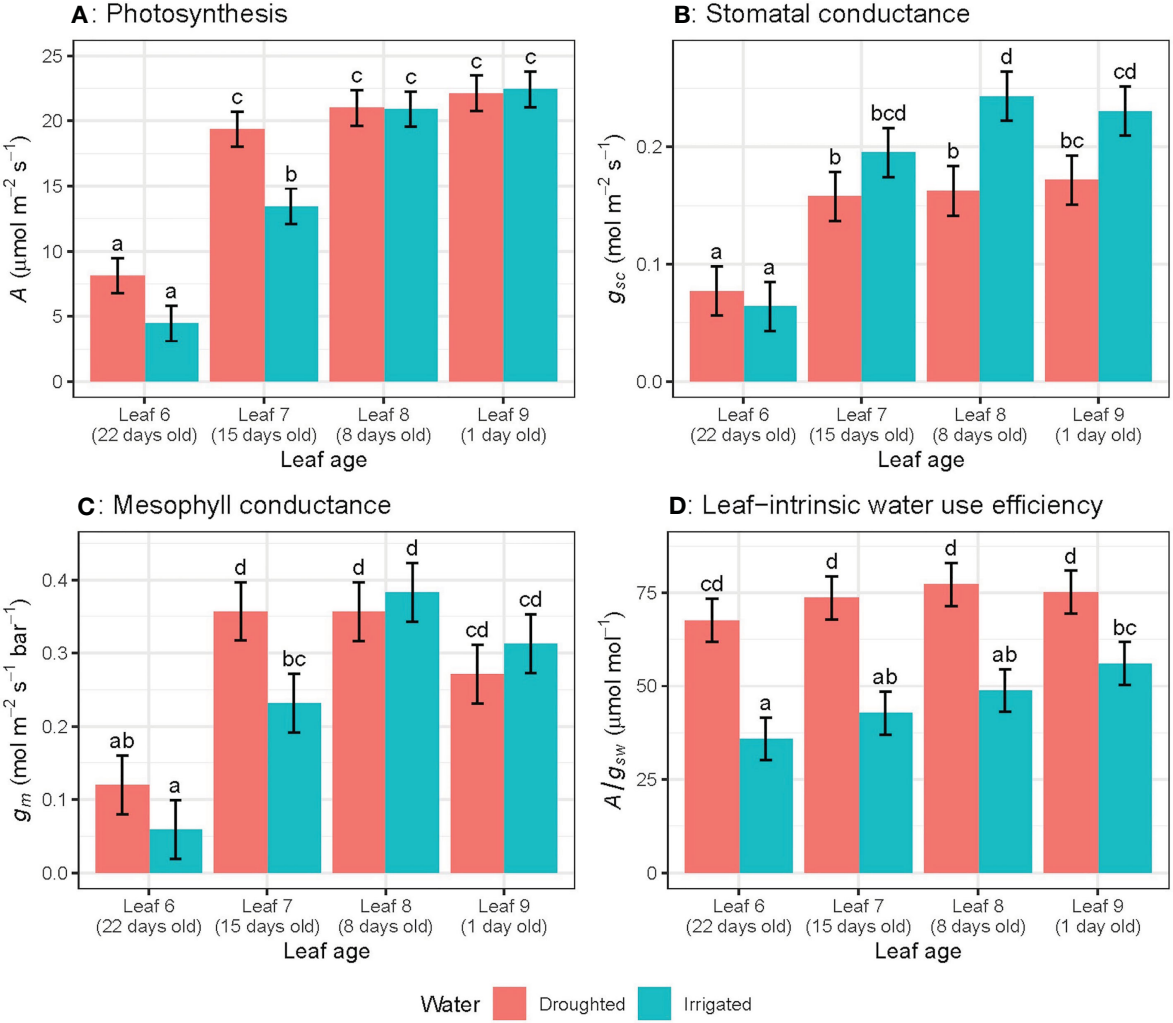
Light-saturated photosynthetic rate (A) **(A)**, stomatal conductance to CO2 (gsc) **(B)**, mesophyll conductance (gm) **(C)** and leaf-intrinsic water use efficiency (A/gsw) **(D)** for wheat leaves of different ages in both well-watered and water-stressed conditions on the ninth week of measurement. Values are mean ± standard error, n = 5. Letters indicate significant differences (P <0.05).

Photosynthetic rate (*A*), stomatal conductance to CO_2_ (*g*_sc_) and mesophyll conductance (*g*_m_) significantly (*P<* 0.01) decreased as leaves aged (**Figures 2A–C**) regardless of water treatments. For leaf 6, *A* decreased by 78% (rate of 0.98 µmol m^–2^ s^–1^ day^-1^), *g*_sc_ by 73% (0.008 mol m^-2^ s^-1^ day^-1^) and *g*_m_ by 87% (0.031 mol m^-2^ s^-1^ bar^-1^ day^-1^) from week 6 to week 9. The linear trend in *A* over the study period differed significantly (*P*< 0.05) among leaf numbers: for leaf 5, the rate of decline was -8.78 ± 4.31 per week, while leaf 6 declined by -4.89 ± 1.34 per week, and leaf 7 declined by -2.06 ± 4.31 per week (**Figure 2A**). This linear trend was also significant (*P*< 0.05) for *g*_sc_ (**Figure 2B**), whereas the linear trend was not significant for *g*_m_ and *A*/*g*_sw_ (**Figures 2C, D**).When leaves of different ages were compared, *A, g*_sc_ and *g*_m_ for the oldest leaves (22 day-old) were significantly lower than younger leaves (15, 8 or 1 day-old) (**Figures 3A–C**). There were no significant leaf age effects for leaf-intrinsic water-use efficiency (*A*/*g*_sw_) (**Figure 2D**), or between different leaves of different ages (**Figure 3D**).

### Plant age effects

**Figure 4:**
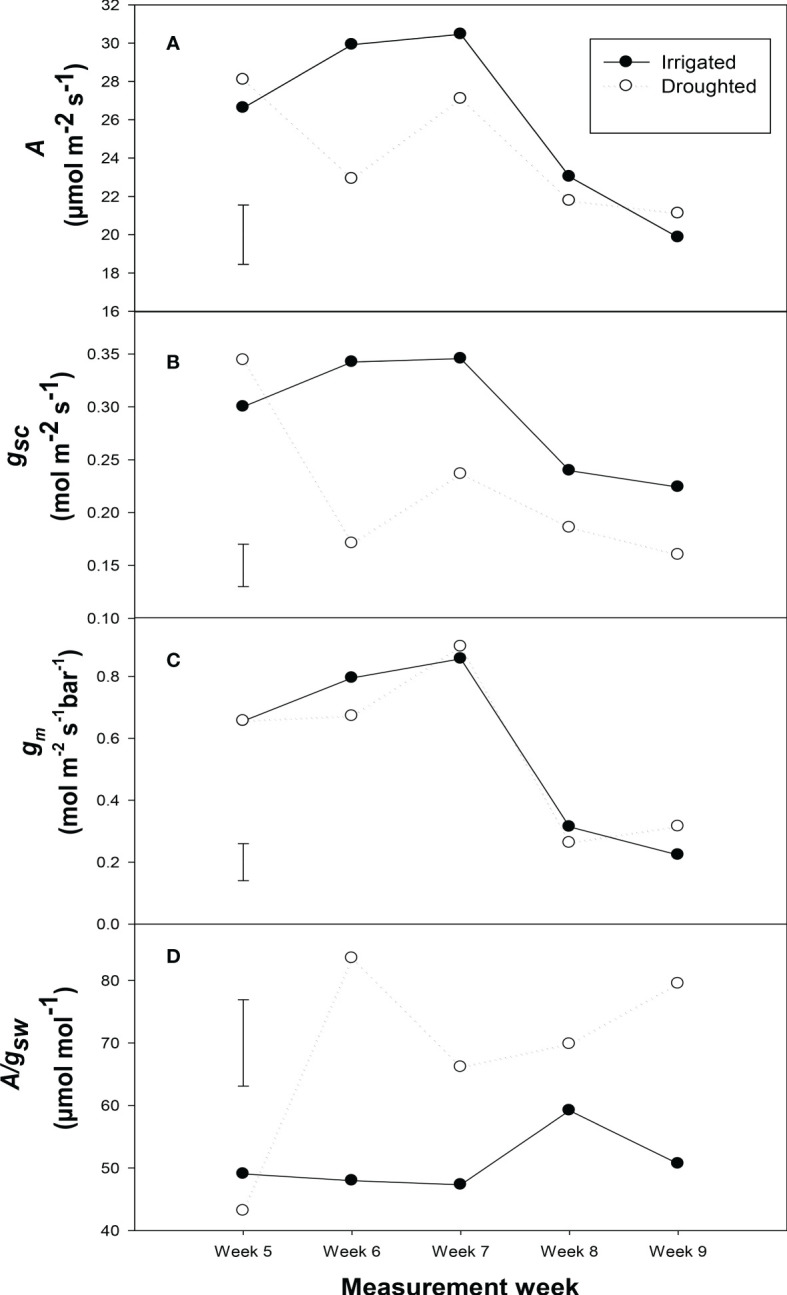
Light-saturated photosynthetic rate (A) **(A)**, stomatal conductance to CO2 (gsc) **(B)**, mesophyll conductance (gm) **(C)** and leaf-intrinsic water use efficiency (A/gsw) **(D)** for the youngest fully expanded wheat leaves at the beginning of each week of measurement in both well-watered and water-stressed conditions. Here the water treatment was started at 6th week of plant age. The youngest fully expanded leaves were measured each week. The bar represents the average standard error, n = 5.

Photosynthetic rate (*A*), stomatal conductance to CO_2_ (*g*_sc_) and mesophyll conductance (*g*_m_) of the youngest fully expanded leaves decreased significantly (*P<* 0.001) from week 5 to week 9, in well-watered plants (**Figures 4A–C**). In well-watered plants, *A*, *g*_sc_ and *g*_m_ of the youngest fully expanded leaf initially increased with rising plant age up to week 7, and then decreased rapidly with increasing plant age. Leaf-intrinsic water-use efficiency (*A*/*g*_sw_) was not affected by plant age for well-watered plants from week 5 to week 9 (**Figure 4D**).

### Water availability effects

The water-stress treatment applied in the second experiment resulted in significantly (*P* = 0.05) more negative midday leaf water potentials (*Ψ*_L_) for water-stressed compared to well-watered plants (averages of -1.4 and -1.1 MPa across all leaf ages for water-stressed and well-watered plants, respectively) (**Supplementary Table 1**).

Only stomatal conductance to CO_2_ (*g*_sc_) was significantly higher (*P* = 0.03) in well-watered plants compared to water-stressed plants, when individual leaves were considered (**Figure 2B**). These changes resulted in higher *A*/*g*_sw_ in drought plants compared to irrigated plants. However, the slope of the decline in photosynthetic rate (*A*) with leaf age did not significantly differ between well-watered and water-stressed plants (**Figure 2A**). Overall, mesophyll conductance (*g*_m_) was slightly, but not significantly, higher in well-watered compared to water-stressed leaves when individual leaves were compared up to week 8 (**Figure 2C**).

When we consider leaves at different ages measured on the same day, water availability did not affect photosynthetic rate for 1 day- and 8 day-old leaves, whereas *A* was significantly (*P* = 0.05) higher for water-stressed plants compared to well-watered plants, for 15 and 22 day-old leaves (**Figure 3A**). However, the rate of decline in photosynthetic rate (*A*) with different leaf positions varied significantly (*P* = 0.03) for well-watered plants only (**Figure 3A**). Well-watered plants showed significantly higher *g*_sc_ compared to water-stressed leaves, only for 1 day- and 8 day-old leaves, whereas for the oldest leaf measured at day 22, water-stressed plants had higher *g*_sc_ than well-watered leaves at the same age. There was no water effect on *g*_m_ for any leaf ages except 15 day-old leaves (**Figure 3C**). Water-stressed plants had significantly higher *A*/*g*_sw_ compared to well-watered leaves for all leaf ages (**Figure 3D**). Moreover, the rate of decline in *A*/*g*_sw_ with different leaf positions varied significantly (*P<* 0.03) for both well-watered and water-stressed plants (**Figure 3D**)

Considering the youngest fully expanded leaf over 5 weeks of measurement, water-stress decreased *A* and *g*_sc_ (*P* = 0.01), but the proportional decrease in *A* was less than the decrease in *g*_sc_, resulting in higher values of *A*/*g*_sw_ (*P<* 0.001) for water-stressed plants compared to well-watered plants. Leaf-intrinsic water-use efficiency increased with increasing plant age (*P* = 0.001) only for water stressed plant (**Figure 4D**). Water-stress did not significantly affect *g*_m_ for the youngest fully expanded leaf at any period.

### Relationship between g_m_ and A, and g_m_ and g_sc_

Significant positive relationships (*P<* 0.001) were found between *g*_m_ and *A*, and between *g*_m_ and *g*_sc_ in both well-watered and water-stressed conditions. The relationship between *g*_m_ and *A* was stronger (well-watered: *g*_m_ = 0.028*A* – 0.078, *r* = 0.78, *P*< 0.001; water-stressed: *g*_m_ = 0.027*A* – 0.095, *r* = 0.58, *P*< 0.001) than between *g*_m_ and *g*_sc_ (well-watered: *g*_m_ = 1.90*g*_sc_ + 0.027, *r* = 0.69, *P*< 0.001; water-stressed: *g*_m_ = 1.59 *g*_sc_ + 0.19, *r* = 0.45, *P*< 0.001). However, there was no significant water availability effect (*P* > 0.05) on the association between *g*_m_ and *A*, or between *g*_m_ and *g*_sc_ (**Supplementary Figure S1**).

### Anatomical properties

**Figure 5:**
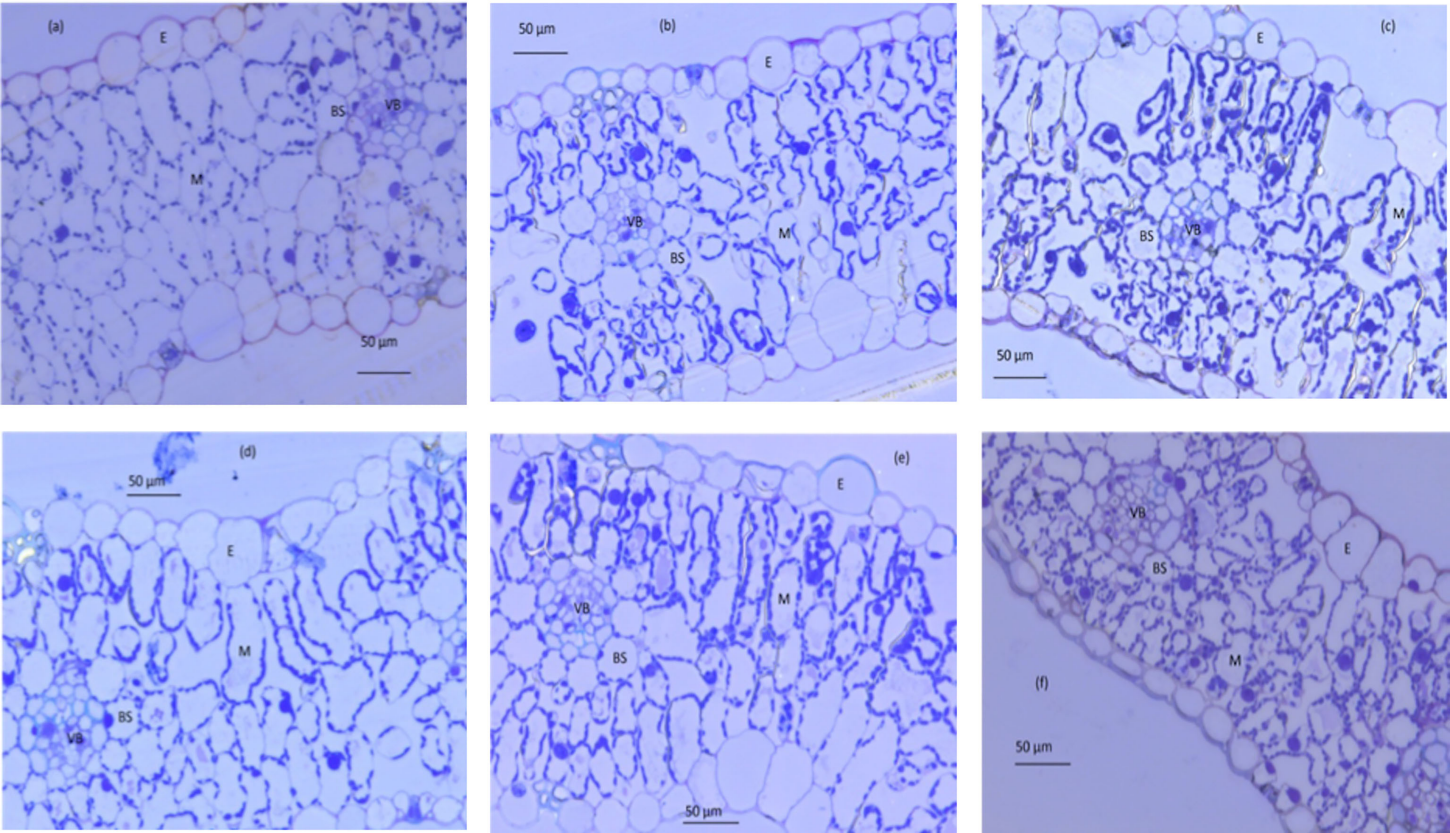
Light micrograph of transverse leaf sections at three different ages (old, mature and young) for well-watered plants (**A–C**, respectively) and water-stressed plants (**D–F**, respectively). BS, outer bundle-sheath cell; E, epidermis; M,mesophyll cell; VB, vascular bundle. Photos are taken at magnification of 400×.

Changes were observed in leaf structure as leaves aged, with young leaves having the highest surface area of chloroplast exposed to intercellular airspace (*S*_c_) (*P*< 0.001), followed by mature leaves, and then old leaves (**Table 1**). The ratio between *S*_c_ and the surface area of mesophyll cells exposed to the intercellular airspaces (*S*_mes_) was significantly higher for young leaves compared to old leaves (*P*< 0.001), whereas there was no age effect on *S*_mes_. Mesophyll thickness was marginally (but not significantly, *P* = 0.08) higher for well-watered compared to water-stressed plants, whereas *S*_c_/*S*_mes_ was higher for water-stressed plants compared to well-watered plants (but not statistically significant, *P* = 0.06) (**Table 1**). *S*_c_ declined more quickly with increasing age for well-watered leaves compared with that for water-stressed leaves (**Figure 5**). Furthermore, the number of chloroplasts per unit leaf surface area was higher for water-stressed plants than for well-watered plants but did not change with leaf age. Taken together, these results suggest that water limitation had subtle effects on chloroplast number, size and distribution, and on the changes in these anatomical properties as leaves aged. Moreover, there was no water or leaf age effect on *f*_ias_ (**Supplementary Table 2**).

**Table 1:** The effect of leaf age (young, mature and old) on leaf thickness (µm), mesophyll thickness (µm), the surface area of mesophyll cells exposed to the intercellular airspaces (Smes), the surface area of chloroplast exposed to intercellular airspace (Sc), The ratio between Sc and Smes and chloroplast number per unit leaf area (Z) for wheat leaves from well-watered and water-stressed plants.

Treatments		Anatomical Parameters					
Leaf age	Water	*Leaf thickness* (µm)	*Mesophyll thickness* (µm)	*S_mes_*	*S_c_*	*S_c_/S_mes_*	*Z* (μm^-2^)
Young	Irrigated	1301 ± 54a	1031 ± 63a	11.3 ± 1.2a	9.0 ± 0.9d	0.80 ± 0.03c	0.70 ± 0.03a
Mature	Irrigated	1350 ± 60a	1050 ± 42a	10.7 ± 0.3a	7.0 ± 0.4bc	0.66 ± 0.04b	0.61 ± 0.07a
Old	Irrigated	990 ± 260a	735 ± 199a	9.5 ± 0.9a	5.0 ± 0.5a	0.52 ± 0.02a	0.60 ± 0.18a
Young	Drought	829 ± 239a	606 ± 179a	9.8 ± 0.9a	8.1 ± 0.5cd	0.82 ± 0.04c	1.03 ± 0.25a
Mature	Drought	1019 ± 277a	776 ± 213a	10.7 ± 0.7a	8.7 ± 0.8cd	0.80 ± 0.03c	0.94 ± 0.28a
Old	Drought	843 ± 376a	623 ± 274a	10.8 ± 1.0a	5.8 ± 0.3ab	0.55 ± 0.05ab	1.07 ± 0.36a

Values are mean ± standard error, n = 4. Letters indicate significant differences (P <0.05).

**Figure 6:**
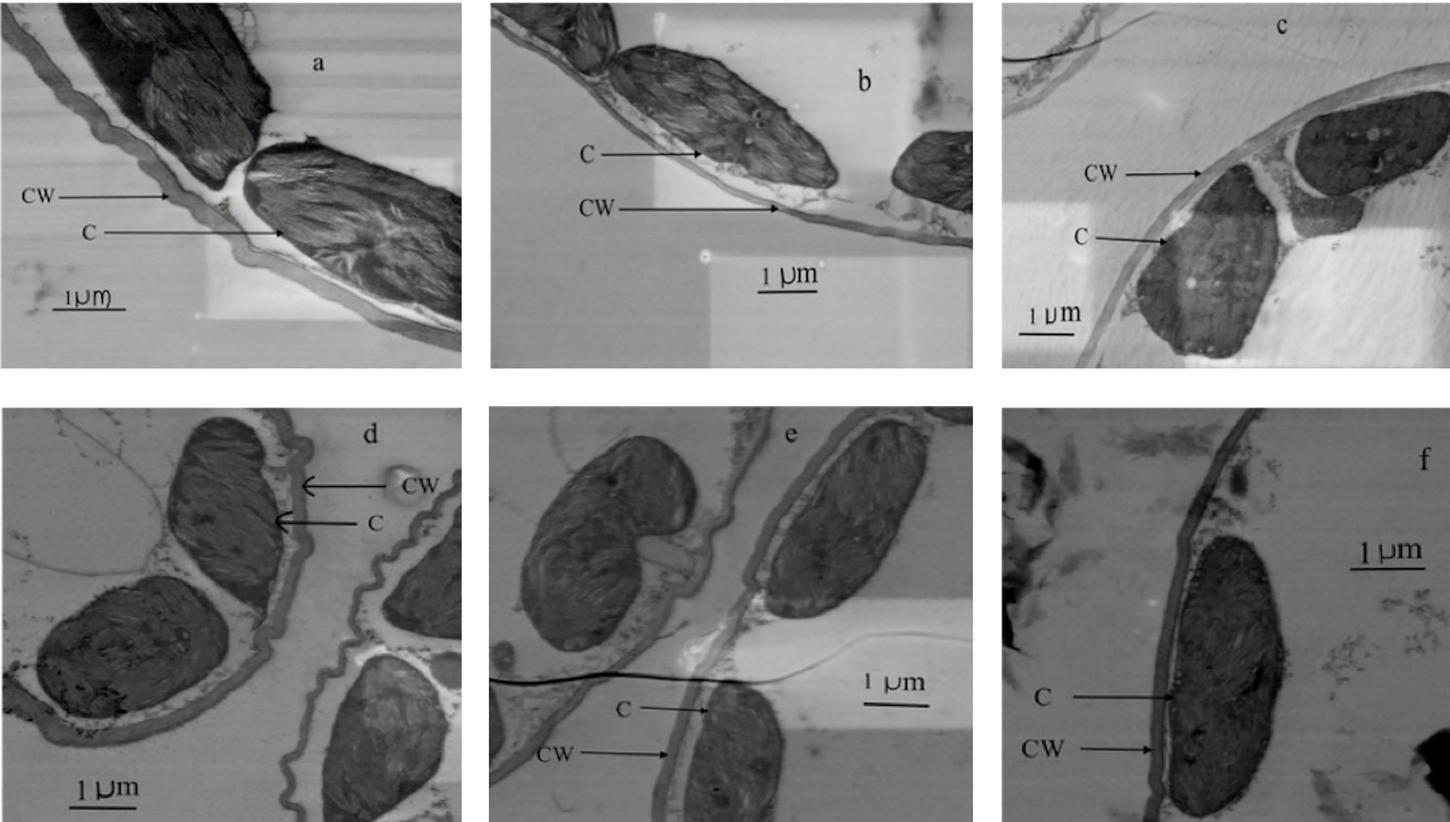
Transmission electron micrographs of transverse leaf sections at three different ages (old, mature and young) for well-watered plants (**A–C**, respectively) and water-stressed plants (**D–F**, respectively). CW, Cell wall thickness; C, Chloroplast.

**Figure 7:**
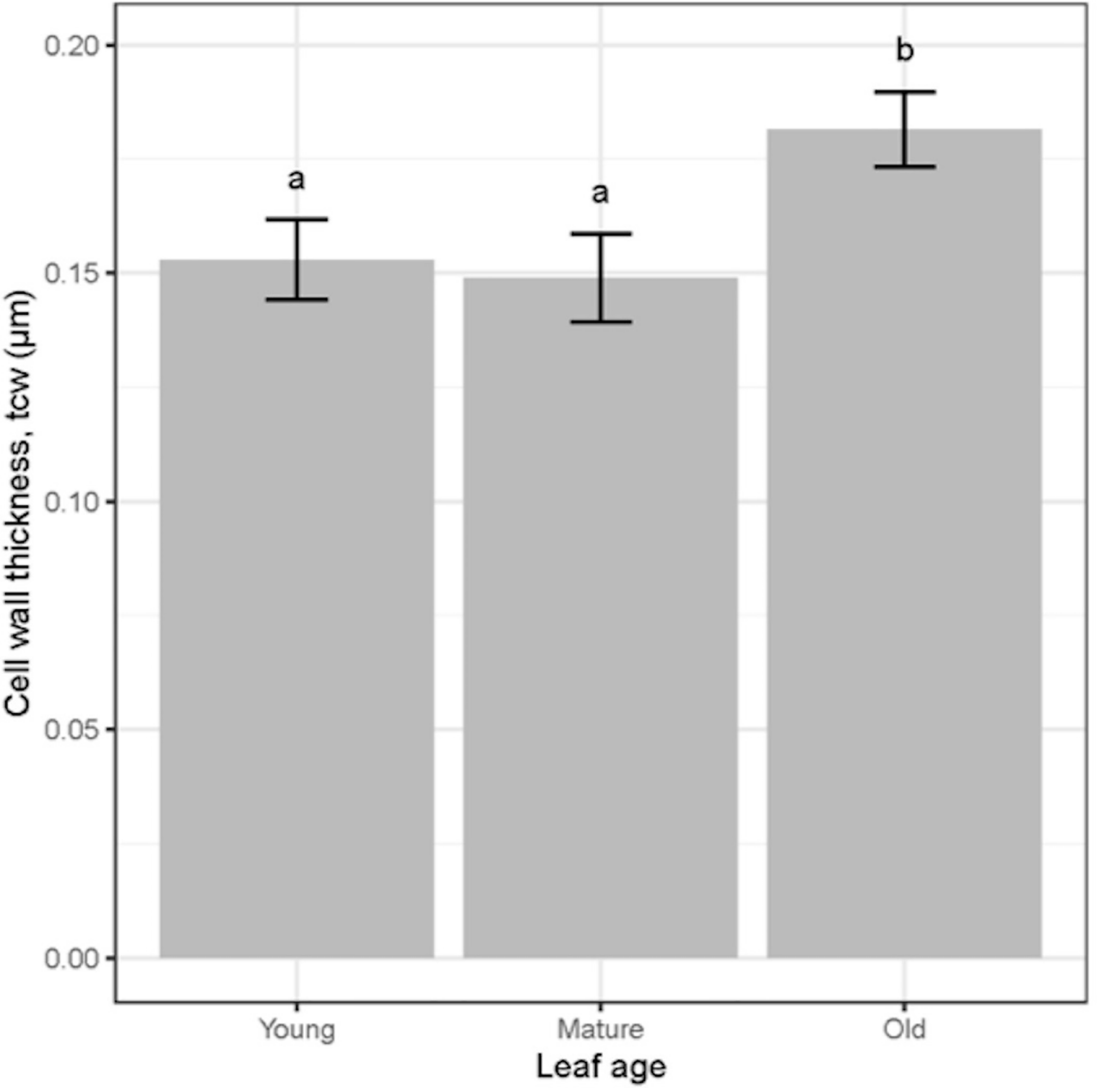
Cell wall thickness (tcw) for wheat leaves of three different ages. Values are mean ± standard error, n = 4. Letters indicate significant differences (P <0.05).

**Figure 8:**
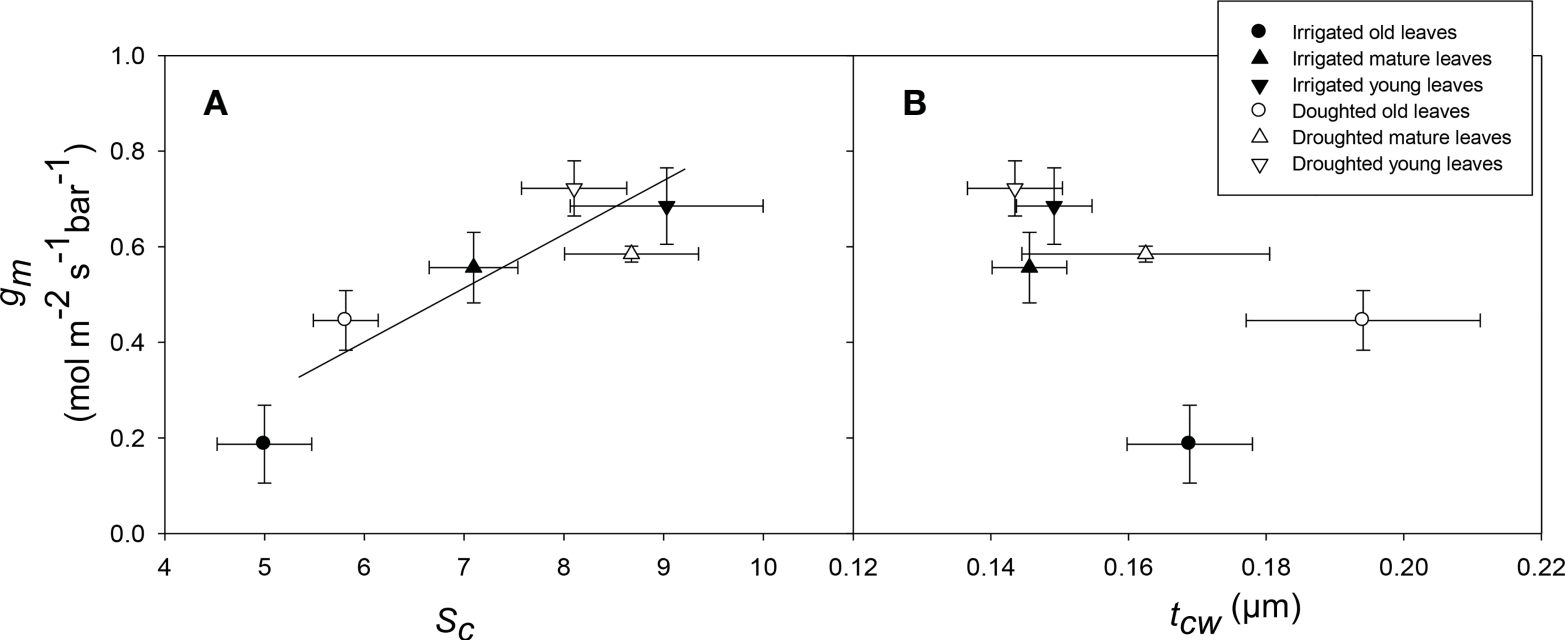
The relationship between surface area of chloroplast exposed to intercellular airspace (Sc) and mesophyll conductance (gm) **(A)**, and mesophyll cell wall thickness (tcw) and mesophyll conductance (gm) **(B)** for wheat leaves at three different ages for well-watered and water-stressed plants. Values are mean ± standard error, n = 4.

There was a significant (*P* = 0.01) relationship between *t*_cw_ and different leaf stages; young leaves had significantly lower *t*_cw_ compared to old leaves regardless of the water treatment (**Figures 6**, **7**). There was a significant positive relationship between *S*_c_ and *g*_m_ (*P* = 0.01) (**Figure 8A**) whereas, the relationship between *t*_cw_ and *g*_m_ was not significant (*P* = 0.22) (**Figure 8B**) for three different leaf stages at two water treatments.

### Re-watering effect

**Figure 9:**
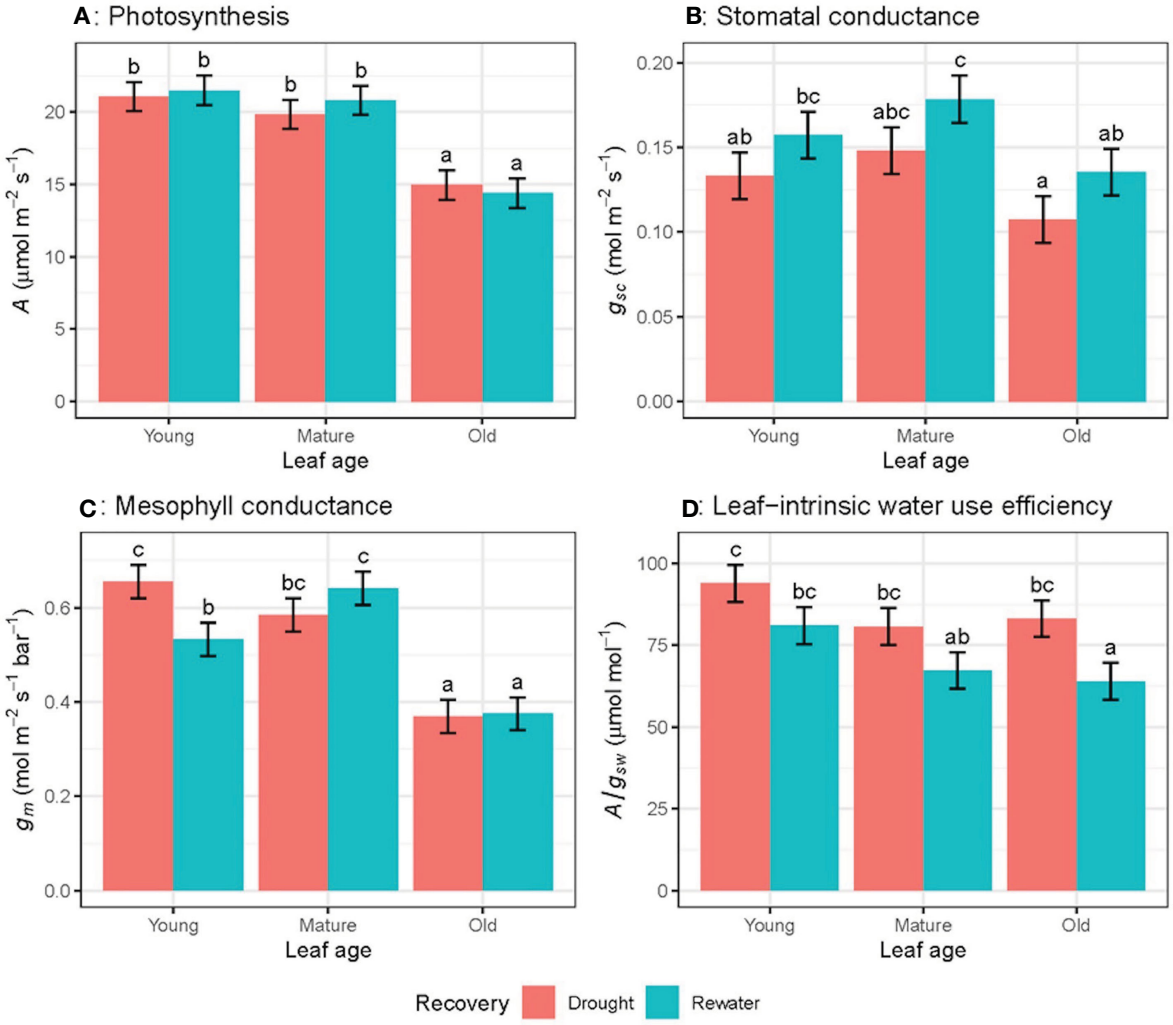
Response of gas exchange parameters for wheat plants remaining under water-stressed conditions and after being rewatered for leaves of differing age for light-saturated photosynthetic rate (A) **(A)**, stomatal conductance to CO2 (gsc) **(B)**, mesophyll conductance (gm) **(C)** and leaf-intrinsic water use efficiency (A/gsw) **(D)**, where the first and second measurements were made 5-8 days apart, n = 4. Letters indicate significant differences (P <0.05).

After one day of re-watering, leaves were less water stressed as *Ψ*_L_ rose from -1.38 MPa to -1.10 MPa. Gas exchange parameters were measured on the same leaves for water-stress plants and one week later following re-watering. As expected, *g*_sc_ significantly increased after re-watering (**Figure 9B**), whereas *A* and *A/g_sw_* did not change significantly following re-watering (**Figures 9A, D**). There was a significant interaction effect between re-watering and leaf age in *g*_m_ (*P* = 0.05) where *g*_m_ decreased for young leaves and increased for mature leaves (**Figure 9C**).

## Discussion

### Mesophyll conductance declines as wheat leaves aged

It is widely observed that stomatal conductance (*g*_sc_) and photosynthetic rate decline as leaves aged and includes crops such as rice (Gu et al. (2012)) and maize (Azizian and Sepaskhah (2014)) and other herbaceous plants (e.g. *Arabidopsis* (Flexas et al. (2007)). Other examples include an alpine orchid (Zhang et al. (2008)) and woody species such as pine (Warren (2006)) and oak (Niinemets et al. (2005)). The decline in *A* has sometimes been attributed to a decline in mesophyll conductance (*g_m_*) in oak (Niinemets et al. (2005), and mustard (Monti et al. (2009)) which in some instances was due to changes in leaf anatomy including tobacco (Clarke et al., 2021), maple (Hanba et al. (2001) and poplar (Tosens et al. (2012)). However, other studies in pine report that *g*_m_ limitations to *A* did not increase with leaf age (Warren (2006)) and in southern beech, *g*_m_ itself was not significantly lower in mature compared to young leaves (Whitehead et al. (2011)). We found that *A* declined by 1.2 µmol m^–2^ s^–1^ day^-1^, and that *g*_m_ declined by 0.035 mol m^-2^ s^-1^ bar^-1^ day^-1^, as leaves matured. Evans and Vellen (1996) also report a decline in *A* and *g*_m_ as wheat leaves aged but did not provide actual ages of the leaves so a direct comparison is not possible. We have previously observed declines in *A* and *g*_m_ of 1 µmol m^–2^ s^–1^ day^-1^ and 0.05 mol m^-2^ s^-1^ bar^-1^ day^-1^, respectively, over one week for newly expanded leaves in the wheat cultivar ‘Scout’, but no significant decline in *A* or *g*_m_ over same the period for the cultivar ‘Cranbrook’ (Jahan et al., 2014). Clarke et al. (2021) observed that *A* and *g*_m_ decreased by 68% and 50% respectively among different leaf positions over 3 weeks. Similarly, we also observed that *A* and *g*_m_ decreased by 64% and 61% respectively among different leaves over the same 3-week period (from **Figure 3**).

Anatomical and structural changes are observed during and after leaf development. When fully mature *Populus tremula* leaves are aging (based on a leaf plastochron index, LPI, of 6 to 12), leaf thickness (*t*_leaf_), mesophyll thickness (*t*_mes_), *S*_mes_ and fraction of internal airspace (*f_i_*_as_) remained constant (Tosens et al., 2012), while *S*_c_, *S*_c_/*S*_mes_, thickness of chloroplasts (*t*_chl_) and length of chloroplasts (*L*_chl_) began to decrease (Tosens et al., 2012). In the current study, we also observed that *S*_c_ and *S*_c_/*S*_mes_ decreased with increasing leaf age, but leaf age did not affect *f*_ias_, and that this was due to a decline in the size of individual chloroplasts for young leaves compared to old leaves. Mesophyll conductance and *S*_c_ were strongly positively correlated in wheat leaves. Similarly, Evans (2020) also observed in his review paper that for any given species, *g*_m_ varies in direct proportion to *S*_c_.

Clearly, *S*_c_ is partly responsible for the reduction in *g*_m_ observed in the current experiment. However, the age-related reduction in *S*_c_ is proportionally much smaller than in *g*_m_, so other anatomical and metabolic factors must also play a role. In general, a negative relationship was found between cell wall thickness (*t*_cw_) and *g*_m_ because of the longer pathway for CO_2_ diffusion from the intercellular airspace to the plasma membrane (Peguero-Pina et al., 2012; Tosens et al., 2012; Giuliani et al., 2013; Peguero-Pina et al., 2015). In this experiment, old leaves have thicker *t*_cw_ compared to young or mature leaves, and overall *g*_m_ is not associated with *t*_cw_; this indicates that across the leaf stages, cell wall thickness is responsible for age-related reduction in *g*_m_ for wheat cultivar ‘Tasman’. Evans (2020) reviewed *g*_m_ in C4 leaves and suggested that the effective porosity of cell walls decrease with increasing cell wall thickness. Hence, changes in the porosity of cell walls with varying leaf age need to be considered to identify the association of *g*_m_ and *t*_cw_ at different leaf ages. In addition to anatomical and structural factors, biochemical factors should be considered in association with *g*_m_ and *t*_cw_ at different leaf ages. For example, Warren (2006) observed that the down-regulated activity of Rubisco with age was responsible for the variation in *g*_m_ with leaf age for *Pinus pinaster*, and for wheat, Rubisco activation decreases in mature leaves compared to old leaves (Sharwood et al., 2016).

Mesophyll conductance can be divided into three components: conductance through intercellular airspaces (*g*_ias_), through cell walls (*g*_w_) and through the liquid phase inside cells (*g*_liq_) (Flexas et al., 2008). In this experiment, gas-phase conductance from sub-stomatal cavities to the outer surface of cell walls (*g*_ias_) was calculated as described by Tosens et al. (2012); we observed no leaf age effect on *g*_ias._ Apart from anatomical changes, enzyme activity also typically declines as leaves age, including carbonic anhydrase, and aquaporins. Carbonic anhydrase (CA) catalyses the reversible interconversion of CO_2_ with HCO_3_^-^, and occurs in multiple forms in the chloroplast, cytosol, mitochondria and plasma membrane (Fabre et al., 2007; Ogeíe et al., 2018). The influence of CA activity on photosynthesis in C_3_ plants is small (Price et al., 1994; Williams et al., 1996), and the effects of CA on *g*_m_ have not been widely reported. Gillon and Yakir (2000) measured both *g*_m_ and total leaf CA activity in tobacco and soy, and reported no relationship between them. However, on a latitudinal genotype transect in *Populus trichocarpa*, northern genotypes exhibited higher *A* as a function of higher *g*_m_ and elevated CA activity (Momayyezi and Guy, 2017). However, it is well known that CA activity declines as leaves age (Novichkova et al., 2006), and therefore the decline in *g*_m_ as leaves age may be in part due to the decline in CA activity.

Aquaporins influence the diffusion of CO_2_ through membranes and may regulate *g*_m_ (Hanba et al., 2004; Flexas et al., 2012; Uehlein et al., 2012; Ermakova et al., 2021). Terashima and Ono (2002) investigated the involvement of aquaporins in CO_2_ diffusion across the plasma membrane of *Vicia faba* and observed that *g*_m_ decreased by 40% and 30% when the leaflets were fed with the aquaporin inhibitor 0.3 mM and 1.2 mM HgCl_2_, respectively. Evans et al. (2009) reviewed the role of aquaporins in CO_2_ diffusion conductance in plants and suggested that aquaporins are responsible for 25% of lipid-phase diffusion conductance. Moreover, Boron et al. (2011) also suggested that aquaporins play a key role in membrane permeability. A wide range of aquaporins are known in plant leaves, with more than 30 isoforms identified in higher plants, and they constitute the most abundant plasma membrane and tonoplast proteins (reviewed by Maurel et al., 2008). The expression of different aquaporins occurs during leaf development (Wei et al., 2007; Prodo and Maurel, 2013), so aquaporin expression and activity may contribute to age-related decline in *g*_m_ although this is yet to be demonstrated. Therefore, high membrane permeability may explain the lack of an association between *g*_m_ and cell wall thickness.

Further studies on membrane permeability and the porosity of cell wall are needed to examine the effects of leaf anatomical characteristics and metabolic factors (aquaporins) for different aged leaves on *g*_m_ in wheat plants. Obtaining a thorough understanding of the association of *g*_m_ with anatomical traits is important to interpret photosynthetic traits, which will lead to a better understanding of how to manipulate *g*_m_ to improve plant performance and increase yield in stressful environments.

### Water-deficit slowed the rate of age-related decline in A and g_m_

A review by Flexas et al. (2007) demonstrated that a reduction in *g*_m_ in response to drought is commonly observed. For example, in *Populus tremula*, water stress reduced the exposed chloroplast to leaf area ratio (*S*_c_/*S*) and increased cell wall thickness, and these changes combined to reduce *g*_m_ (Tosens et al., 2012). However, reduced *g*_m_ in response to drought is not always observed (Flexas et al., 2012), or is transient (Flexas et al., 2009; Galle et al., 2009). In the current experiment, one week after the water treatment started (week 6), water-deficit plants had slightly lower *g*_m_ (0.53 mol m^-2^ s^-1^ bar^-1^) than irrigated plants (0.58 mol m^-2^ s^-1^ bar^-1^) averaged across all leaf ages. Three weeks later, these same leaves of water-deficit plants (now 15- and 22-day-old leaves) had higher *g*_m_ and *A* than their well-watered counterparts. That is, the reduction in *g*_m_ and *A* as leaves aged was less pronounced in water-deficit plants than well-watered, perhaps reflecting a slowing of leaf development under water-deficit.

### 4.3 Leaf age affected the response to rewatering

Wheat plants re-watered after a water stress event showed an increase in *g*_sc_ and different *g*_m_ recovery rates for different aged leaves, which indicates that photosynthetic machinery is preserved with no irreversible damage. Water stress has been implicated in reducing photosynthetic biochemistry such as maximum photochemical efficiency (F_v_/F_m_) and the efficiency of electron transfer through PSII (Φ_PSII_), however, rewatering for 14 days restored Fv/Fm and Φ_PSII_ to pre-water deficit conditions (Wang et al., 2018). Mesophyll conductance (*g*_m_) was somewhat reversible when water stress was released in sugar beet (Monti et al., 2006) while Galle et al. (2009) observed a rapid recovery of *g*_m_ in tobacco from water stress in Spring compared with Summer. Peguero-Pina et al. (2018) observed complete recovery of *g*_m_ in holm oak (*Quercus ilex* L.) after re-watering. Furthermore, it has been showed that *g*_m_ recovered more quickly in *Eucalyptus dumosa* than in *Eucalyptus pauciflora* because aquaporins were deactivated in *E. pauciflora* associated with higher oxidative stress (Cano et al., 2014). In this study, rates of *g*_m_ recovery for different leaf ages were not equal when comparing water-deficit plants and plants after re-watering. That is, young leaves did not recover to unstressed values after re-watering, while in mature leaves *g*_m_ increased after re-watering. There is no clear explanation for the differences in rates of recovery after rewatering, but these differences may relate to: a) the conditions under which the leaves formed (young leaves formed under water stress, while mature leaves did not); b) differences in leaf anatomy including maturity of stomata development (the degree of recovery of stomatal conductance was inversely related to leaf age); or c) redistribution of resources from old leaves to younger leaves.

We consider the applied water stress as moderate because when measured in high light and moderate vapour pressure deficit, stomatal conductance to water vapour (*g*_sw_) was higher than 0.16 mol H_2_O m^-2^ s^-1^ and the average leaf water potential (*Ψ*_L_) was -1.38 MPa (**Supplementary Table 2**.). Similarly, Shrestha et al. (2018) also observed similar stomatal conductance to water vapour (*g*_sw_) for three chickpea genotypes and described them as moderately water stressed. Leaf age, and/or duration of water stress can alter the recovery rate of leaf physiological parameters; e.g. Warren et al. (2011) found that long-term water stress delayed the recovery rate of *A* in *Eucalyptus*. Genotypic variation was also found to affect the recovery rate of *A* between two *Eucalyptus* species (Cano et al., 2014).

## Conclusion

We found that gas exchange parameters, including mesophyll conductance, declined as wheat leaves aged, but that the rate of decline was reduced when plants were moderately water stressed. The rate of recovery after rewatering water-stressed plants varied between leaves of differing ages. In terms of anatomical changes with physiological parameters, the surface area of chloroplasts exposed to intercellular airspaces (*S*_c_) declined as leaves aged, resulting in a positive correlation between *g*_m_ and *S*_c_. In addition, cell wall thickness (*t*_cw_) increased for old leaves compared to mature/young leaves; however, the association between *g*_m_ and *t*_cw_ was not significant. Therefore, in addition to high *S*_c_ and low *t*_cw_, high membrane permeability could explain higher *g*_m_ for young leaves.

## Data availability statement

The original contributions presented in the study are included in the article/**Supplementary Material**. Further inquiries can be directed to the corresponding author.

## Author contributions

EJ: Conceptualization, designed and conducted the experiment including technical measurements and analysis of data, developed initial draft of manuscript. RS: Provided suggestion and feedback on and edited the manuscript. DT: Provided feedback on the overall design of the study, results and drafts of the manuscript, specifically critical review, commentary and editing. All authors contributed to the article and approved the submitted version.
