# Supplementary material for: Effects of leaf age during drought and recovery on photosynthesis, mesophyll conductance and leaf anatomy in wheat leaves
Source: Front Plant Sci. 2023 Jun 20;14:1091418. doi: 10.3389/fpls.2023.1091418 (PMC10318540; doi:10.3389/fpls.2023.1091418)
Supplement: Supplementary file 1 [file DataSheet_1.docx]

Fig. S1. The relationships between mesophyll conductance (*g*_m_) and light-saturated photosynthetic rate (*A*), and between mesophyll conductance (*g*_m_) and stomatal conductance to CO_2_ (*g*_sc_), in both irrigated (closed symbols) and water-stressed (open symbols) conditions.

Data were modelled by linear regressions; A: (Irrigated) *g*_m_ = .028*A* - 0.078, *r* = 0.78, *P* < 0.001 & (Drought) *g*_m_ = .027*A* - 0.095, *r* = 0.58, *P* < 0.001 B: (Irrigated) *g*_m_ = 1.90*g*_sc_ + 0.027, *r* = 0.69, *P* < 0.001 & (Drought) *g*_m_ = 1.59 *g*_sc_ + 0.19, *r* = 0.45, *P* < 0.001.

**Table S1.** The average leaf water potential **(***Ψ*_L_) for water-stressed and well-watered leaves.

| *Water treatments* | *Leaf water potential* (*Ψ*_L_) |
| --- | --- |
| Water-stressed | -1.38 ± 0.09 MPa |
| Well-watered | -1.10 ± 0.11 Mpa |

**Table S2.** The average volume fraction of inter-cellular air space (*fi*as) for well-watered and water-stressed leaves.

| *Water treatments* | Volume fraction of inter-cellular air space (*fi*as) |
| --- | --- |
| Water-stressed | 0.35 ± 0.01 |
| Well-watered | 0.34± 0.01 |
